# Challenging case of deficient mismatch repair right-sided locally advanced adenocarcinoma of the ascending colon with duodenal involvement: A case report including step-by-step video of operation

**DOI:** 10.1016/j.ijscr.2023.109137

**Published:** 2023-12-12

**Authors:** Alexander A.J. Grüter, Malaika S. Vlug, Ide T. Spaanderman, Adriaan D. Bins, Tineke E. Buffart, Jurriaan B. Tuynman

**Affiliations:** a Amsterdam UMC location Vrije Universiteit Amsterdam, Department of Surgery, De Boelelaan 1117, Amsterdam, the Netherlands; b Cancer Center Amsterdam, Treatment and quality of life, Amsterdam, the Netherlands; c Amsterdam UMC, Department of Medical Oncology, De Boelelaan 1117, Amsterdam, the Netherlands

**Keywords:** Right-sided colon cancer, Right hemicolectomy, Neoadjuvant therapy, Deficient mismatch repair, Locally advanced cancer, Case report

## Abstract

**Introduction and importance:**

Irresectable colon cancer presents a complex clinical challenge. Neoadjuvant immunotherapy has shown potential in improving resectability. Additionally, advancements in surgical techniques, including complete mesocolic excision (CME) with central vascular ligation (CVL), have contributed to better outcomes for right-sided colon cancer. This case report aims to demonstrate the successful laparoscopic resection of initial appearing irresectable colon cancer with suspected duodenal involvement.

**Case presentation:**

A 70-year-old female presented with an irresectable mismatch repair deficient (dMMR) adenocarcinoma of the ascending colon with suspected duodenal ingrowth. Neoadjuvant treatment with pembrolizumab and ataluren resulted in a significant response, allowing for surgical resection. A laparoscopic right hemicolectomy with CME, including CVL, intracorporeal anastomosis and extraction through a Pfannenstiel incision, was performed. Additionally, the serosal layer of the duodenum was shaved after observing the absence of intraluminal invasion. Postoperatively, transient gastroparesis occurred, but overall outcomes were favourable.

**Clinical discussion:**

This case emphasizes the potential of immunotherapy in improving resectability for irresectable dMMR colon cancer with suspected involvement of surrounding organs. The combination of neoadjuvant therapy and advanced surgical techniques, such as CME with CVL, shows promise in achieving favourable clinical outcomes. However, further studies are needed to validate the effectiveness and safety of this combined approach in a larger cohort of patients.

**Conclusion:**

The successful laparoscopic resection of initially irresectable dMMR colon cancer with duodenal involvement, following neoadjuvant immunotherapy, demonstrated promising outcomes. This case advocates for further exploration of neoadjuvant treatments' efficacy, coupled with advanced surgical techniques, in managing locally advanced right-sided colon cancer.

## Highlights

• Approximately 10–20 % of colorectal cancer cases demonstrate deficient mismatch repair
• Immunotherapy can improve resectability in some locally advanced colorectal tumors
• The techniques in right hemicolectomy have significantly evolved over the decades
• The innovations include for example CME and the intracorporeal anastomosis
• The application of these innovations appears to yield improved clinical outcomes

## 1 Introduction

Colon cancer is a prevalent malignancy, particularly in elderly patients. In some cases, tumors can become irresectable due to local invasions into surrounding organs. Despite advances in surgical techniques and systemic therapies, the management of irresectable colon cancer remains a complex and often unsatisfactory endeavor [1]. Neoadjuvant treatment approaches have emerged as promising strategies to improve the resectability of locally advanced colon cancer, offering potential benefits in terms of tumor downsizing, reducing the extent of surgery, and increasing the likelihood of achieving negative surgical margins. The FOxTROT trial has provided compelling evidence supporting the efficacy of neoadjuvant chemotherapy in improving surgical outcomes for locally advanced colon cancer [2]. Furthermore, an additional dimension to neoadjuvant therapy involves immunotherapy, particularly noteworthy in cases of deficient mismatch repair (dMMR) related colon cancer [[3], [4], [5]]. Studies indicate that approximately 10–20 % of colorectal carcinoma cases exhibit dMMR [6]. This leads to impaired DNA repair mechanisms, leading to increased neoantigens and thus sensitivity to immunotherapy. These therapies aim to exploit specific immune checkpoints to unleash an effective antitumor immune response or disrupt aberrant cellular signaling pathways critical for tumor growth and survival.

In addition to advancements in systemic therapies, innovations within the realm of surgical techniques have also played a pivotal role in improving outcomes for patients with colon cancer. For example, in the field of the right hemicolectomy, a variety of novel approaches have been introduced. These include the adoption of intracorporeal anastomosis, extraction via the Pfannenstiel incision, and the implementation of complete mesocolic excision (CME) along with central vascular ligation (CVL) and D2 lymphadenectomy. The superior mesenteric vein (SMV) has become a crucial organ within this surgical procedure by performing CME, including CVL and D2 lymphadenectomy. All these newer approaches hold the potential to contribute to improved clinical outcomes [[7], [8], [9]].

This case report presents the management and step-by-step laparoscopic resection of an initial appearing irresectable ascending colon carcinoma with suspected duodenal involvement. The aim of this paper is to provide a comprehensive and detailed overview of the surgical challenging procedure through the use of a video demonstration, including details of the case and clinical outcomes of this specific patient.

This case has been documented in accordance with the enhanced SCARE checklist. The SCARE Guidelines were introduced in 2016 to establish a standardized framework for reporting surgical case studies. Subsequently, in 2018, a refined and improved version of the SCARE checklist was introduced following the completion of a Delphi consensus process [10].

## 2 Case presentation

**Fig. 1:**
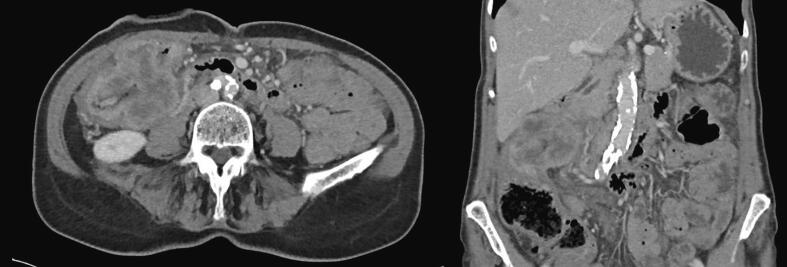
The CT scan before neoadjuvant therapy that shows an ascending colon tumor with suspected ingrowth into the duodenum and abdominal wall.

**Fig. 2:**
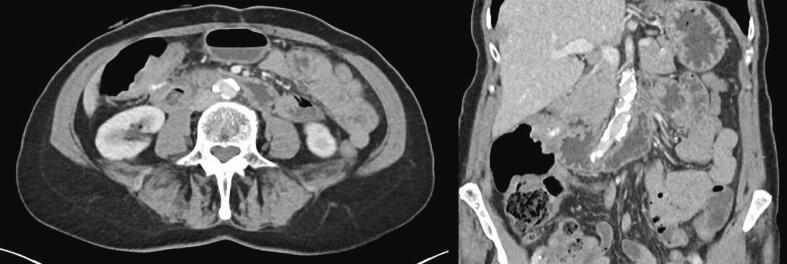
The CT scan following neoadjuvant treatment that demonstrates a remarkable response compared to the CT scan prior to this treatment.

A 70-year-old female patient presented with abdominal pain and weight loss. Colonoscopy revealed an obstructive right-sided tumor. Pathology results showed a dMMR adenocarcinoma (MLH1 promoter hypermethylation) and the CEA was 2.5, indicating that the patient was not a CEA secretor. A subsequent CT scan demonstrated an irresectable ascending colon carcinoma with suspected ingrowth in the duodenum and abdominal wall, without any distant metastasis. See Fig. 1 for this CT scan. Because of the irresectability, neoadjuvant treatment with pembrolizumab (200 mg iv once every 3 weeks) and ataluren (3 times daily; 500 mg, 500 mg and 1250 mg), in the phase 2 ATAPEMBRO study, was started. In preparation for potential complications from obstruction, a loop ileostomy was created. After 5 cycles of neoadjuvant treatment, the patient developed an acute kidney injury and liver enzyme disorder, leading to the discontinuation of the treatment. A new CT scan showed an impressive response to the neoadjuvant therapy, making the patient a candidate for surgical resection. In Fig. 2, this CT scan is displayed. At this point, the CEA was still not elevated and had a value of 4.

The video demonstrates a step-by-step surgical procedure performed on this patient: a laparoscopic right hemicolectomy including shaving and closure of the duodenum. A medial-to-lateral approach was utilized to access the submesenteric plane. The CME technique, including D2 lymphadenectomy and CVL, was performed; the SMV was fully exposed until the gastrocolic trunk of Henle and the ileocolic vessels were ligated at their origin. During surgery, the tumor was found to be adhesive to the duodenum, making it challenging to distinguish between fibrosis/inflammation and tumor. Based on a pretreatment gastroscopy, which showed no intraluminal invasion of the duodenum, and the significant response observed on the CT scan, a decision was made to resect the tumor, including only the serosal layer of the duodenum. The serosal layer of the duodenum was closed crosswise, and a side-to-side intracorporeal anastomosis between the ileum and transverse colon was performed (see Table 1 for a detailed step-by-step description of the procedure).

Postoperatively, the patient experienced gastroparesis, for which a nasogastric tube was placed and total parental feeding was initiated. There were no other complications. The patient was discharged after 9 days and underwent follow-up according to the guidelines of the ATAPEMBRO trial. Pathology results showed a ypT0N1c tumor with 18 negative lymph nodes and 1 tumor deposit (dMMR). Follow-up CT scans at 3, 6 and 9 months postoperatively showed no signs of recurrence or metastasis, and the CEA remained below 5 throughout this follow-up.

**Table 1:** Step-by-step procedure of the laparoscopic right hemicolectomy with shaving of the duodenum.

Step 1	Closure ileostomy and setup and exposure of operating field.
Step 2	Submesenteric dissection through the ileal mesentery (medial-to-lateral).
Step 3	Superior mesenteric vein dissection and ligation of ileocolic vessels.
Step 4	Proximal dissection of the superior mesenteric vein and dissection of Gastrocolic Trunk of Henle.
Step 5	Ligation of right branches of middle colic vessels.
Step 6	Hepatic flexure mobilization and colon and ileum transection.
Step 7	Shaving and closure of duodenum.
Step 8	Intracorporeal side-to-side stapled anastomosis.

## 3 Discussion

The presented case highlights several important aspects in the management of irresectable colon cancer with suspected duodenal involvement. Neoadjuvant therapies, particularly immunotherapy like pembrolizumab, have shown promise in improving resectability and clinical outcomes for patients with locally advanced dMMR colon cancer [[3], [4], [5]]. The significant response observed in this case after neoadjuvant treatment supports the potential role of immunotherapy in downstaging tumors, enabling subsequent surgical intervention. Similar findings have been reported in other studies, further supporting the value of incorporating immunotherapy in the multimodal treatment of locally advanced dMMR colorectal cancer [4,11,12].

Furthermore, advancements in surgical techniques have also contributed to the evolving landscape of colon cancer management. In particular, the introduction of various innovative approaches within the right hemicolectomy procedure, such as intracorporeal anastomosis, extraction via the Pfannenstiel incision, and the application of CME with CVL and D2 lymphadenectomy, represent significant strides in improving clinical outcomes for patients with right-sided colon cancer [[7], [8], [9]]. In the Netherlands, a large-scale national study, called the Right study, is currently being conducted to implement the most optimal minimally invasive right hemicolectomy [13]. This technique incorporates all the aforementioned aspects, along with maintaining a low intra-abdominal pressure (IAP), which has been shown to contribute to improved short-term outcomes as well [14]. The combination of all these aspects holds the potential to lead to the best clinical outcomes for patients with right-sided colon cancer.

The integration of CME with CVL and D2 lymphadenectomy within the right hemicolectomy procedure is particularly noteworthy, as it allows for a more extensive and precise dissection of the mesocolon and mesenteric vessels. By targeting the central vascular structures and extensive lymphadenectomy, potential sources of tumor recurrence and metastasis are effectively addressed, leading to improved oncological outcomes and similar postoperative short-term outcomes, making it an attractive option for the surgical management of advanced colon cancer cases [7,15].

Moreover, the video demonstration of the laparoscopic right hemicolectomy procedure in this case report provides valuable insights for the surgical community. The step-by-step approach, along with the specific details of the surgical challenges encountered, enhances the understanding and replicability of the procedure for other surgeons, contributing to the dissemination of knowledge and improvement in surgical skills.

Despite the success of the presented case, some limitations should be acknowledged. The response to neoadjuvant therapy and subsequent resectability may vary among patients, depending on individual tumor biology and host factors. For instance, extant literature posits that immunotherapy may hold considerable promise for patients with dMMR. Nevertheless, it is important to note that the existing body of research has not yet yielded definitive conclusions on this matter, and as of now, chemotherapy remains the established standard of care for patients afflicted with dMMR locally advanced colorectal carcinoma. Notwithstanding, emerging evidence suggests that dMMR tumors exhibit reduced responsiveness to chemotherapy, thereby potentially underscoring the prospective significance of immunotherapy in the forthcoming landscape [4,16,17]. Hence, further investigation with larger cohorts and long-term follow-up is necessary to validate the effectiveness and safety of the combined approach of neoadjuvant therapy and advanced surgical techniques in irresectable colon cancer cases. Currently, the ATAPEMBRO study is being conducted to investigate the effects of pembrolizumab and ataluren in patients with metastatic or irresectable locally advanced dMMR and proficient colorectal adenocarcinoma, as well as dMMR endometrial carcinoma or stomach carcinoma or small bowel carcinoma [18].

## 4 Conclusion

This case highlights the successful laparoscopic resection of a first appeared irresectable ascending colon carcinoma with suspected duodenal involvement, following neoadjuvant treatment with pembrolizumab and ataluren. The patient achieved a favourable outcome with no signs of recurrence or metastasis during follow-up evaluations at 3, 6 and 9 months. Further studies are warranted to explore the efficacy of neoadjuvant treatments in these types of cases and the added value of this optimal surgical technique including low IAP, CME with CVL and D2 lymphadenectomy, intracorporeal anastomosis and extraction of specimen via Pfannenstiel incision for patients with right-sided colon cancer.

The following is the supplementary data related to this article.

Supplementary material file 1 Video of challenging case of a deficient mismatch repair right-sided locally advanced adenocarcinoma with duodenal involvement. Supplementary material file 1

## CRediT authorship contribution statement

A.A.J. Grüter wrote the paper. M.S. Vlug and J.B. Tuynman gave their feedback on the paper and performed the surgical procedure. I.T. Spaanderman, A.D. Bins and T.E. Buffart gave also their feedback on the paper and were involved in the neoadjuvant treatment of this patient.

## Informed consent

Written informed consent was obtained from the patient for publication and any accompanying images. A copy of the written consent is available for review by the Editor-in-Chief of this journal on request.

## Funding

This research did not receive any specific grant from funding agencies in the public, commercial, or not-for-profit sectors.

## Ethical approval

The ethics committee of Amsterdam UMC, VUmc location, has granted an exemption for this study, because the design is a case report. Of course, written consent has been requested from the patient, and the patient has provided consent for the use of clinical data and video recordings of the surgery. A copy of the written consent is available for review by the Editor-in-Chief of this journal on request.

## Consent

Written informed consent was obtained from the patient for publication and any accompanying images. A copy of the written consent is available for review by the Editor-in-Chief of this journal on request.

## Registration of research studies

Not applicable, design is a case report.

## Guarantor

J.B. Tuynman (email address: j.tuynman@amsterdamumc.nl).

## Declaration of competing interest

The authors declare there are no conflicts of interest.

## Data Availability

My manuscript has no associated data.
